# Ultrasound-Guided Pigtail Catheter Drainage: An Effective Alternative to Exploratory Laparotomy

**DOI:** 10.7759/cureus.33479

**Published:** 2023-01-07

**Authors:** Vikas Jadhav, Chirag R Patel, Rupa M Kopparthi, Rajesh Kuber, Janapamala V S Kishore

**Affiliations:** 1 Radiodiagnosis, Dr. DY Patil Medical College, Hospital & Research Centre, Pune, IND; 2 Radiology, Dr. DY Patil Medical College, Hospital & Research Centre, Pune, IND; 3 Orthopedics, Dr. DY Patil Medical College, Hospital & Research Centre, Pune, IND

**Keywords:** intra-abdominal collections, percutaneous continuous drainage, ultrasonography, interventional radiology, pigtail

## Abstract

Introduction

It has been long established that open surgeries were the only options available for the management of intra-abdominal abscesses or collections. These were associated with increased morbidity and mortality. Traditionally, the idea of percutaneous needling could not gain popularity due to poor localization of collections. However, with the advent of ultrasound, percutaneous pigtail-catheter drainage has proven to be minimally invasive and allows precise localization of the drainage site.

Objectives

To study the effectiveness of ultrasound-guided pigtail catheter drainage as an alternative to exploratory laparotomy for the management of intra-abdominal abscesses or collections.

Materials and methods

A total of 48 patient cases, which included liver abscesses, perinephric collections, malignant ascites, splenic collections, pseudocysts, and psoas abscesses, were studied prospectively in a medical college in India from October 2020 to October 2021. The efficacy of the drainage was assessed by serial ultrasound.

Results

Out of 48 patients, 34 were male and 14 were female, ranging in age from 19 to 64 years, who were diagnosed with intra-abdominal abscesses or collections and underwent ultrasound-guided pigtail catheter drainage. The average hospital stay for patients was 2.5 days. They were followed up periodically for three months post-procedure, and none had significant complications or recurrence.

Conclusion

The pigtail catheter is the treatment of choice for liquefied intra-abdominal collections or abscesses, which helps to reduce post-procedure hospital stays and complications.

Contribution

This article reiterates the use of minimally invasive techniques in place of open surgeries with less morbidity.

## Introduction

Intra-abdominal collections are one of the major groups of morbidities experienced by the medical and surgical teams. Usually, small unilocular collections resolve spontaneously with medical therapy alone, without any surgical interventions. However, large abscesses or collections usually require surgical interventions like laparotomies for their treatment. For a long period, patients were subjected to extensive surgeries for the management of intra-abdominal abscesses or collections. These were associated with increased morbidity, mortality, and complications such as adhesions, as well as an increased hospital stay, which further exposes a patient to the risk of infections. Hence, for better patient care, it is vital to avoid major surgical procedures. Percutaneous therapeutic procedures like pigtail catheter insertion have been increasingly performed nowadays as an alternative to open surgical laparotomies [1,2,3].

Traditionally, the idea of percutaneous needle aspiration could not gain popularity due to the poor localization of collections. However, with the advent of ultrasound, percutaneous pigtail catheter drainage has proven to be safe, effective, minimally invasive, and allows precise localization of the drainage site. Percutaneous drainage has been used for liver abscesses for more than six decades now. However, these procedures had high failure rates due to their poor localization. Later, Gerzof et al. proved that percutaneous drainage was effective when used under the guidance of ultrasound and computed tomography together [2]. Eventually, Haaga and Weinstein showed that the safety and effectiveness of these procedures were better under computed tomography guidance [3]. Drainage procedures done under computed tomography (CT) guidance have the disadvantage of radiation exposure. In this study, we aim to demonstrate that ultrasound-guided pigtail catheter drainage can be safely, effectively, and precisely used for intra-abdominal collections. This article abstract has been accepted by the 19th Asian-Oceanian Congress of Radiology (AOCR) 2021 Scientific Committee for publication in the Korean Journal of Radiology Supplementary Issue (ISI Journal, JCR Impact Factor 3.730). Later, it was withdrawn as it was only a supplementary issue and not the main issue.

## Materials and methods

It is a descriptive interventional study design. A total of 48 patients were studied prospectively at the Dr. DY Patil Medical College and Research Center, Pune, from October 2020 to October 2021. Research Protocol No. IESC/PGS/2020/1731 has been approved by the Institutional Ethics Review Board.

All the procedures were carried out on Siemens or Sonosite ultrasound machines, as per their availability. Written consent was taken, and all the patients were informed about the technique and possible complications before the procedure. Patients were requested for platelet counts, prothrombin time (PT), and international normalised ratio (INR) results before the procedure as a precautionary measure to rule out coagulopathy, which was excluded from the study. Also, solid collections and those with multiple thick internal septations were excluded from the study. The surgical team with immediate operative capability was kept on standby in case of any complications or failure. Abdominal abscesses are typically ellipsoid, displacing surrounding viscera to provide a safe window for percutaneous drainage. None of the collection drainages was refused due to a lack of a safe entry route. The exact percutaneous entry route was meticulously planned on sonography before needling, considering various criteria like size, location, distance from the skin surface, and the anatomic relation of the collection to the surrounding vital structures like vessels and viscera. Proper sterile precautions were taken before the procedure. A sterile cover was placed over the ultrasound transducer. A sterile gel was applied over the cover, and the entry route was reconfirmed. As a local anaesthetic, 5 cc of 1% lidocaine was injected subcutaneously. The skin incision was made with an 11-number scalpel blade. Under real-time ultrasound guidance, an 8-14 Fr pigtail catheter was inserted through the desired route, avoiding vital structures like vessels and viscera. The catheter was unscrewed from the troche and advanced into the collection site. The initial sample was collected and sent for diagnostic fluid analysis and antibiotic sensitivity. A three-way stopcock and extension tubing with vacuum drainage bottles were attached. The catheter was fixed externally by suturing it to the skin and placing a piece of tape around the catheter. Patients were monitored post-procedure and underwent CT screening to look for intra-abdominal complications like bleeding, viscus perforation, and catheter placement. No complication was experienced by any of the patients, and all were discharged two to three days post-procedure. Patients were followed up periodically for three months, and none had a recurrence of significant complications.

## Results

Table 1 shows the proportion of individual causes of abscesses in 48 patients. The efficacy of the drainage was assessed by serial ultrasound. Post-catheter insertion, screening of the abdomen was done using computed tomography to confirm the catheter placement and rule out intra-abdominal bleeding.

**Table 1:** Number of patients treated with ultrasound-guided pigtail catheter drainage in different locations

Location of collection/abscess	Number of patients	Percentage(%)
Liver	18	37.5%
Renal	12	25%
Ascites (Intra-peritoneal)	6	12.5%
Spleen	6	12.5%
Psoas muscle	4	8.3%
Pancreatic pseudocyst	2	4.1%

Out of 48 patients, 34 were male and 14 were female, ranging in age from 19 to 56 years, of which 28 were between 40 and 56 years old. This correlation suggested that older patients were less likely to resolve spontaneously with medical therapy and thus needed interventional procedures.

Patients complained of various symptoms depending on the pathology, most commonly pain in the abdomen (46 patients) and fever (44 patients), generalised weakness (34 patients), pallor (28 patients), jaundice (16 patients), and vomiting (15 patients).

Table 2 shows minor complications like obstruction of the catheter, kinking, and accidental removal or displacement of the catheter tip that were experienced in our study and were effectively managed without the need for an open laparotomy by the surgical team.

**Table 2:** Various complications experienced during our study

Complications	Number of patients	Method of management
Kinking or obstruction of the catheter	2	Saline flushing of the catheter
Accidental withdrawal/ displacement of the catheter tip	2	Repositioning of the catheter under aseptic precautions

## Discussion

This was a prospective study, where the patients with intra-abdominal collections or abscesses were assessed and drained using a pigtail catheter under ultrasound guidance. The treatment of these collections has been dramatically improved, with a significant decrease in mortality and morbidity due to the concurrent use of antibiotics and imaging guidance instead of open surgical drainage [1]. Our study aimed to demonstrate the efficacy of catheter drainage, not only for patients with liver abscesses but also for other intra-abdominal collections like hydronephrosis, malignant ascites, splenic abscesses, psoas abscesses, and large pseudocysts. We also demonstrated that using proper technique, ultrasound guidance was sufficient for percutaneous catheter insertion, and the hazards of radiation from CT can be alleviated.

The overall success rate without any complications in our study was reported to be 91.6%. However, 8.4% of patients experienced minor complications like kinking of the catheter, catheter displacement or removal, and blockage of the catheter due to extremely thick fluid, flakes, and debris. These complications were overcome by repositioning the catheter under aseptic precautions, using larger French-size catheters, and frequent flushing of the catheter with normal saline in cases of obstruction.

**Figure 1:**
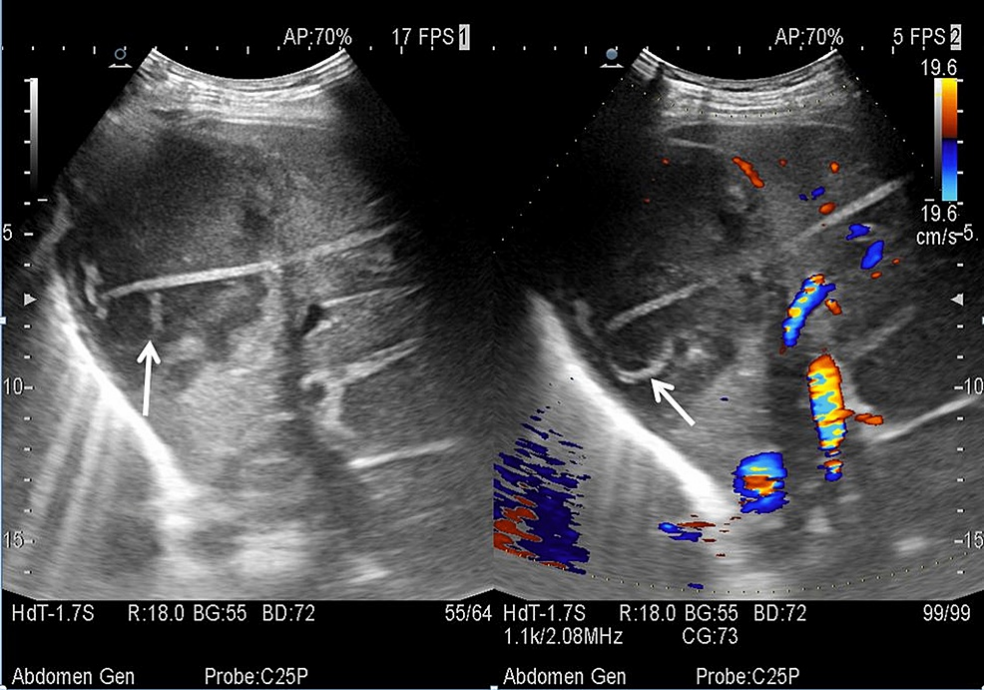
An ill-defined hypoechoic abscess with mild peripheral vascularity was noted in the right lobe of the liver. The tip of the pigtail can be noted in situ (white arrow).

Percutaneous drainage either using needle aspiration or a catheter has proven to be standard management for the treatment of liver abscesses [4]. However, the need for repeated aspirations using a needle had its disadvantage, and hence, a pigtail catheter is considered a better treatment option for liver abscess. (Figure 1) shows a pigtail catheter in situ in a liquefied abscess cavity in the liver, which was removed after three to four days following the collapse of the abscess cavity. In our study, we drained 18 liver abscesses using ultrasound guidance for percutaneous drainage, with 16 of the lesions noted in the right lobe of the liver. All abscess cavities were drained successfully without any complications or need for surgical laparotomy.

**Figure 2:**
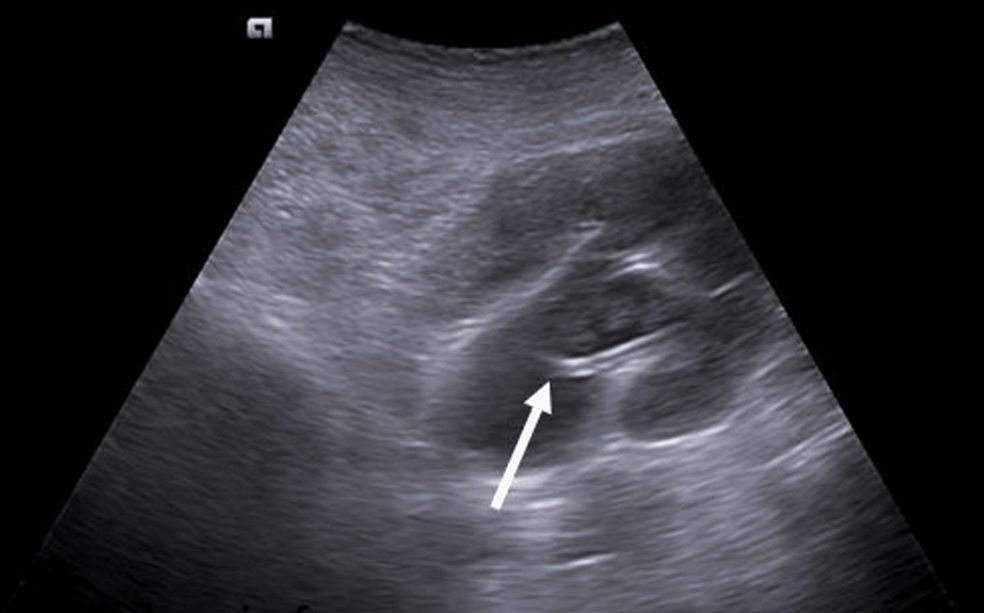
Grade III hydronephrosis for impacted ureteric calculus is noted in the right kidney, with a pigtail catheter noted in situ (white arrow) in the pelvicalyceal system.

Precaution should be taken to avoid any adjacent visceral perforations. Percutaneous nephrostomy (PCN) has proven to be fundamental for upper urinary diversion in cases of hydronephrosis due to impacted ureteric calculus, pyonephrosis, stricture ureter, failed Double-J (DJ) stent, distal ureteral malignancy, etc. Pigtail catheter for direct PCN tube placement is easy to perform under expert hands, precise, and comparatively economical to its counterpart, the wire-guided technique [5]. The potential major complications of PCN are infection, visceral perforation, urinoma, and perirenal hematoma [6]. However, in our study, we encountered two patients with minor complications. One of the patients had accidental catheter displacement, and another experienced kinking of the catheter, both of which were rectified by repositioning the catheter. Figure 2 shows the tip of the pigtail catheter in situ, within the dilated pelvicalyceal system.

**Figure 3:**
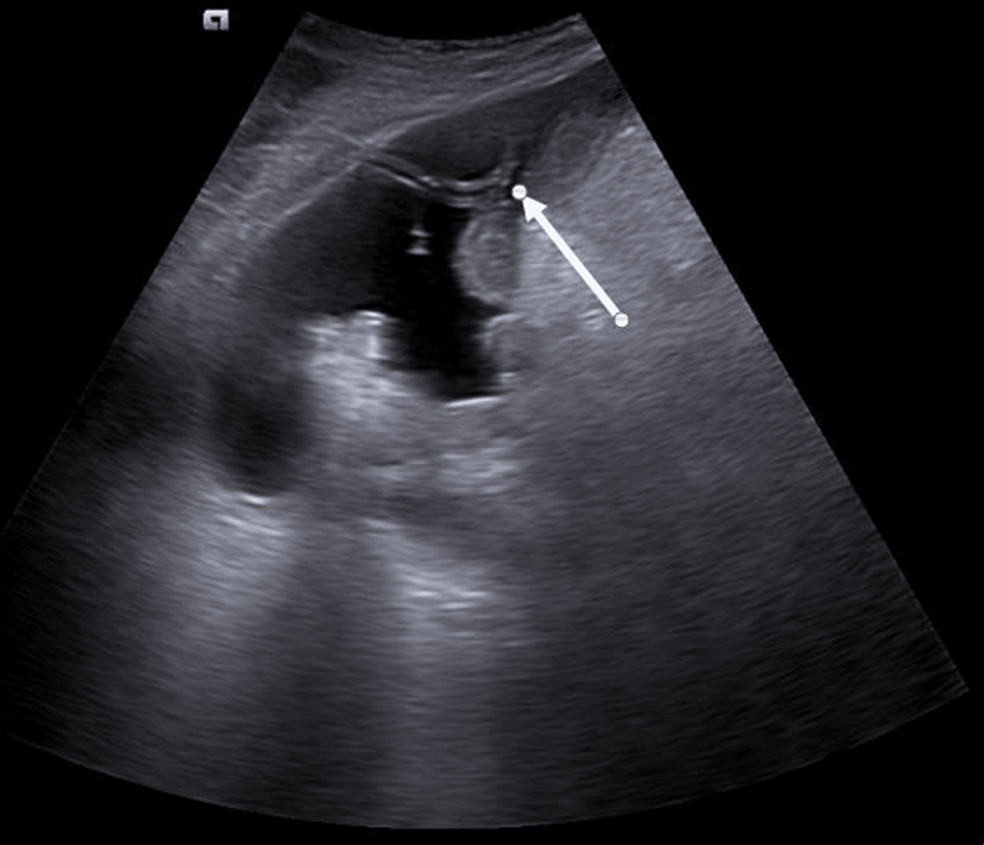
Gross ascites were noted in a known case of stomach carcinoma. A pigtail catheter was inserted (white arrow) for the long-term management of this patient's malignant ascites.

Patients with advanced malignancies and associated refractory ascites often have limited treatment options and a poor prognosis. Also, these subgroups of patients suffer from debilitating symptoms of abdominal distension, early satiety, breathlessness, and vomiting, which further contribute to the impairment of quality of life. Paracentesis has proven to be effective in palliative management; however, it tends to recur, and there is a need for repeated procedures that can cause further complications like infection and viscus perforation [7, 8]. Major cardiovascular complications were avoided by the use of colloids when required and by monitoring the pace of drainage. The interposition of a three-way stopcock between the collection tube and catheter is also effortless. Figure 3 shows an indwelling pigtail catheter in situ providing continuous peritoneal drainage in a case of stomach carcinoma.

**Figure 4:**
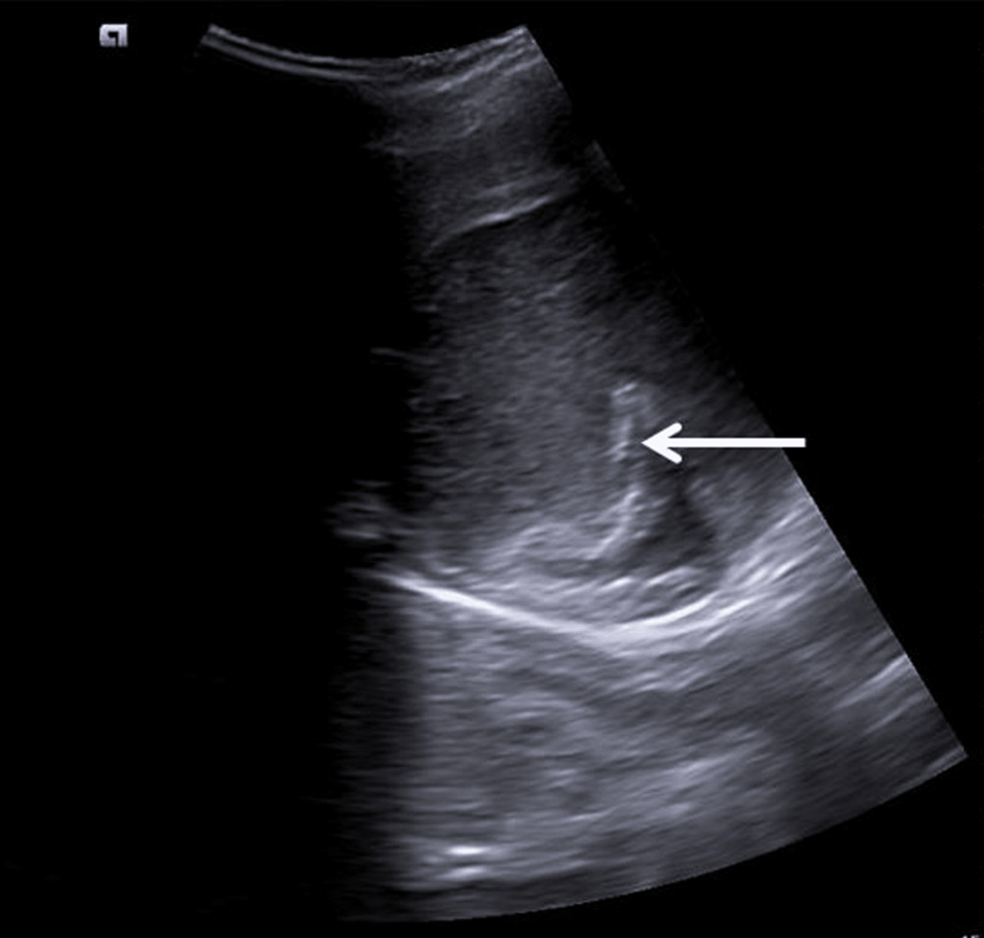
An ill-defined, heterogeneously hypoechoic abscess cavity is noted within the splenic parenchyma. The tip of the pigtail catheter is within the abscess cavity (white arrow).

Splenic abscess presents with vague or nonspecific symptoms, and it is often seen in patients with comorbidities like immunocompromised status [9, 10]. It is a trend among many surgeons that splenectomy is the treatment of choice for splenic abscesses [11]. However, imaging-guided percutaneous drainage of a splenic abscess has proven to be the linchpin for splenic abscess management [12, 13]. Given the fact that many of these patients suffer from comorbidities, this has been established as standard management for splenic abscess. Figure 4 shows an abscess cavity within the splenic parenchyma, which was drained under ultrasound guidance with a pigtail catheter.

**Figure 5:**
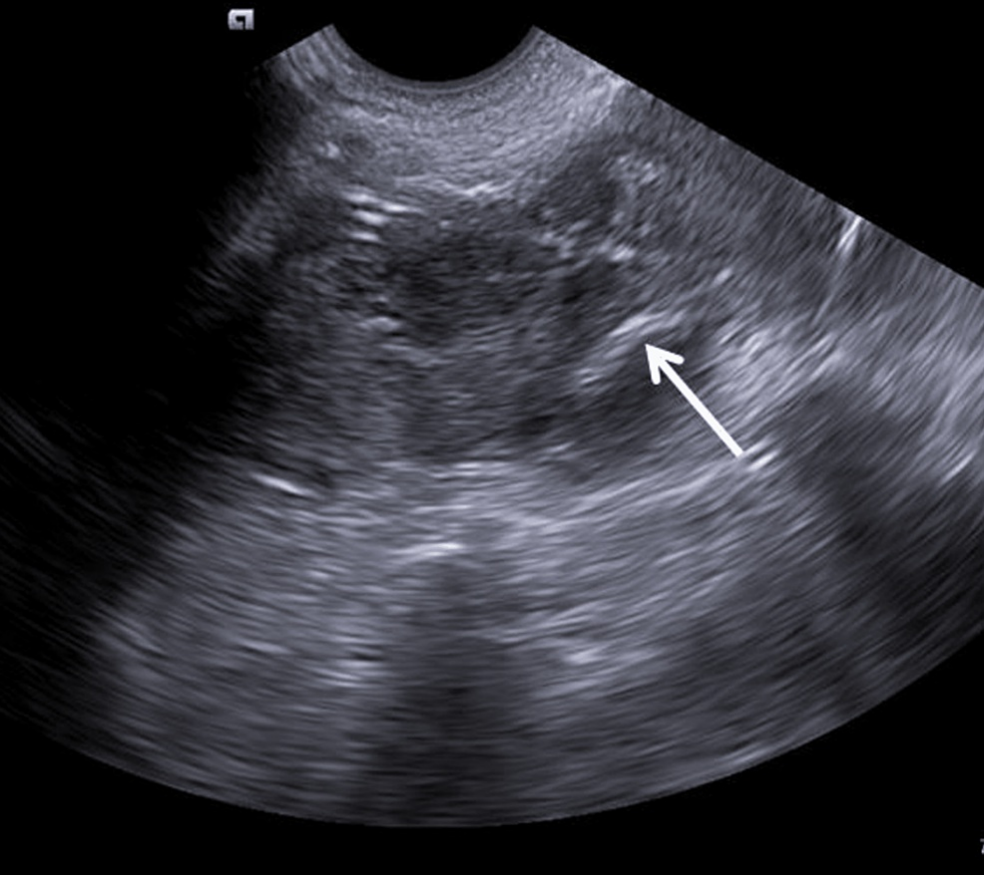
An ill-defined heterogeneously hypoechoic abscess is noted in the left psoas muscle, with the tip of the pigtail catheter noted in situ (white arrow).

In our study, one patient experienced blockage of the catheter after 24 hours of insertion due to extremely thick viscous fluid, large flakes, and debris; this was managed by flushing the catheter with normal saline. Psoas abscess presents with a variable-sized abscess, which may sometimes extend to the entire length of the muscle. Many surgeons advocate open surgical management in psoas abscesses, given that it reduces the pressure within the abscess cavity and relieves the symptoms early [14]. However, this is a major procedure with increased morbidity and complications like sinus formation [15]. In our study, four patients underwent ultrasound-guided percutaneous catheter drainage, and the abscess cavity was drained in all cases. However, one patient experienced obstruction along with displacement of the catheter, which was repositioned, and drainage was continued. Figure 5 shows the tip of the pigtail catheter within the abscess cavity.

**Figure 6:**
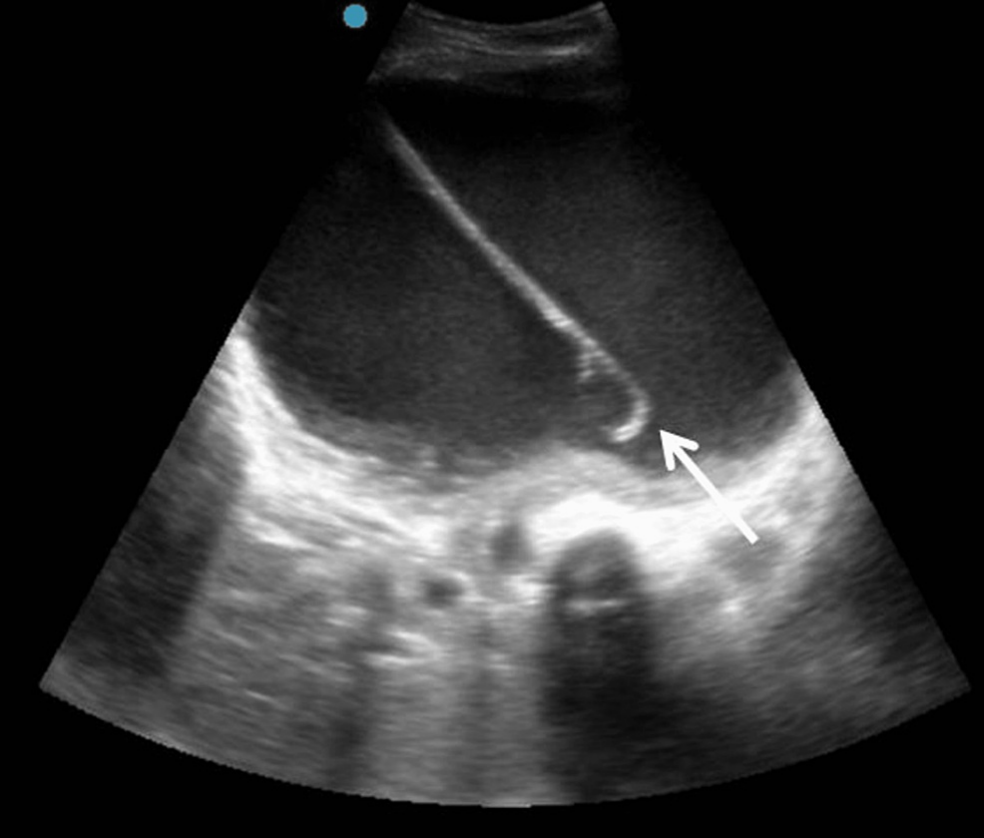
A large, well-defined anechoic collection with internal moving echoes noted arising from the body of the pancreas is suggestive of a pseudocyst. Note the pigtail catheter in situ (white arrow) within the pseudocyst.

Pancreatic pseudocysts are a common complication occurring in cases of acute pancreatitis. External drainage of the pseudocyst is indicated in cases where the pseudocyst is large (more than 5 cm) or when its contents are purulent. However, the pancreatic duct communicates with the pseudocyst, and hence the drainage of the pseudocyst will lead to the refilling of the sac. Recently, surgical exploration using laparoscopy has been introduced; however, it requires general anaesthesia, which is also detrimental and may cause morbidity [16]. Therefore, a continuous drainage system is needed for its management, which is achieved by a percutaneous pigtail catheter. The use of ultrasound guidance for its insertion is of paramount importance as the pancreatic pseudocyst is a retroperitoneal structure, and it is vital to avoid any vascular or visceral damage. Figure 6 demonstrates the tip of the pigtail catheter within a large pancreatic pseudocyst. None of these patients had immediate or chronic complications as a result of pigtail catheter drainage; however, one patient with tuberculosis experienced a recurrence of a psoas abscess due to non-compliance with treatment with AKT.

All patients drained successfully with no major complications, and the catheter was removed after the complete collapse of the cavity, which was seen usually within nine days. None of the patients had any recurrence or delayed complications like fistula formation in their follow-up screening using ultrasound, which was done over a period of three months.

Limitations

We acknowledge the limited sample size of each subgroup and the scope for further dedicated studies. A large number of samples for individual subgroups of collections will help broaden our view of rare complications that may have been missed.

## Conclusions

We concluded from our study that, using a precise technique for pigtail catheter insertion under ultrasound guidance, most intra-abdominal collections can be managed without the need for an exploratory laparotomy. Reduced cost, morbidity, complications, hospital stay, and chances of hospital-acquired infection were drastically reduced.
